# Awareness Status of Schistosomiasis among School-Aged Students in Two Schools on Pemba Island, Zanzibar: A Cross-Sectional Study

**DOI:** 10.3390/ijerph20010582

**Published:** 2022-12-29

**Authors:** Yiyun Liu, Wenjun Hu, Juma Saleh, Yuyan Wang, Qingkai Xue, Hongchu Wu, Kun Yang, Yuzheng Huang

**Affiliations:** 1 School of Public Health, Nanjing Medical University, Nanjing 211166, China; 2 National Health Commission Key Laboratory of Parasitic Disease Control and Prevention, Jiangsu Provincial Key Laboratory on Parasite and Vector Control Technology, Jiangsu Institute of Parasitic Diseases, Wuxi 214064, China; 3 Tropical Diseases Research Center, Nanjing Medical University, Wuxi 214064, China; 4 Neglected Diseases Program, Ministry of Health, Zanzibar 999132, Tanzania

**Keywords:** knowledge, attitude and practice, schistosomiasis, Zanzibar

## Abstract

Schistosomiasis elimination has been set as a target in the Neglected Tropical Disease Roadmap of 2021 to 2030. The present study assessed the level of understanding, awareness and behaviors of schistosomiasis among students in Zanzibar and explored the influencing factors as the basis for reliable suggestions for the follow-up policy on schistosomiasis prevention and control. A Knowledge, Attitude and Practices (KAP) survey on students’ perceptions of schistosomiasis was conducted on students from grades 4–9 at two selected schools on Pemba, Zanzibar, from May through September in 2021. A total of 217 valid participants responded to the questionnaires. T-test and chi-squared tests were used to examine the association between the dependent and explanatory variables. Multiple linear regressions were used to analyze the influencing factors of KAP. The findings indicated a lack of knowledge about schistosomiasis among the participants. Although respondents were aware of the risks of infection, they continued to engage in high-risk activities. Age, family size and presence of hematuria were found as contributing factors. Elder students performed better on knowledge (*p* = 0.02) and attitude (*p* < 0.01) scores, and students with a smaller family received higher attitude scores (*p* = 0.04). Practice was significantly correlated with gender (*p* < 0.01) and hematuria (*p* < 0.01). Several kinds of health education should be adopted to raise students’ basic knowledge of schistosomiasis. It is also critical to make the community aware regarding schistosomiasis. Future efforts for the prevention and control of schistosomiasis should employ an integrated strategy combining communities with schools to encourage behavioral change.

## 1. Introduction

Schistosomiasis is a neglected worldwide tropical disease caused by trematode flukes of the genus *Schistosoma* [1]. It is considered to be a major public health problem in tropical and subtropical regions, especially in African countries [2]. Zanzibar is a part of Tanzania and consists of two main islands, Unguja and Pemba, with many ponds and streams, where residents are susceptible to urogenital schistosomiasis caused by *Schistosoma haematobium* via freshwater contact [3]. Two rainy seasons from May to June and November to December, and the average annual temperature ranges between 23 °C and 28 °C in Pemba [4]. Pemba on average receives more rainfall than Unguja, and there are many ponds and streams on the island especially during the rainy season; roads, low-lying areas and ponds are more prone to overflowing and flooding, causing residents more vulnerable to infection [5,6,7,8,9]. The main economic activities include seawater fishing and cash crop production [4]. School-aged children are at the highest risk for parasite infections, because of more frequent water-contact behavior such as swimming and playing in the water. In addition, in school-aged children, chronic schistosomiasis can also cause iron-deficiency anemia, growth retardation and malnutrition, leading to cognitive impairment [10]. Hence, World Health Organization (WHO) recommends school-aged children who live around ponds and streams should be focused to become a research population for baseline surveys and monitoring and evaluation of intervention strategies [11].

Currently, mass drug administration (MDA) is a key strategy for schistosomiasis control recommended by the WHO [12]. An estimated 16.5 million people in Tanzania required preventive chemotherapy in 2020, of whom 40.9% were school-aged children [13,14]. Additional improvements may be attained from multi-sectoral collaboration, including increasing access to safe water, improving sanitation, and hygiene (WASH), implementing snail control, behavioral changes and health education [15]. In 2014, WHO signed a tripartite memorandum of understanding (MOU) with China and Zanzibar, paving the way for the launch of a pilot project to eliminate schistosomiasis in Zanzibar. Integrative strategies for schistosomiasis control with human treatment, snail management, and behavior interventions have been implemented on Pemba from 2017 to 2020. The prevalence in Uwandani with more than 2500 residents decreased from 8.92% to 0.64% after two years of intervention [4,16]. The elimination of schistosomiasis from a public health perspective can be achieved with appropriate control strategies, as demonstrated by the practice on Pemba Island. However, COVID-19 lasting nearly three years, unsustainable access to MDA, uncontrolled spreading of the *Bullinus* snail and insufficient health education have postponed the progress to achieving the 2030 goal of schistosomiasis elimination issued in the NTD Roadmap 2030.

Many studies have shown that monitoring and evaluating the distribution of schistosomiasis and community knowledge attitudes and practices play an important role in sustainable control interventions. Therefore, focusing on sustainable schistosomiasis control, students’ cognitive level and understanding of the disease need to be assessed, which might provide guidance for policy makers. Therefore, as the first Schistosomiasis-related KAP study conducted by a Chinese project team in Zanzibar, the purpose of this study is to describe the knowledge, attitude and practice of pupils in grades 5–9 and explore the influencing factors towards schistosomiasis in order to schedule relevant health education and necessary control measures.

## 2. Methods

### 2.1. Study Design and Public Involvement

**Figure 1:**
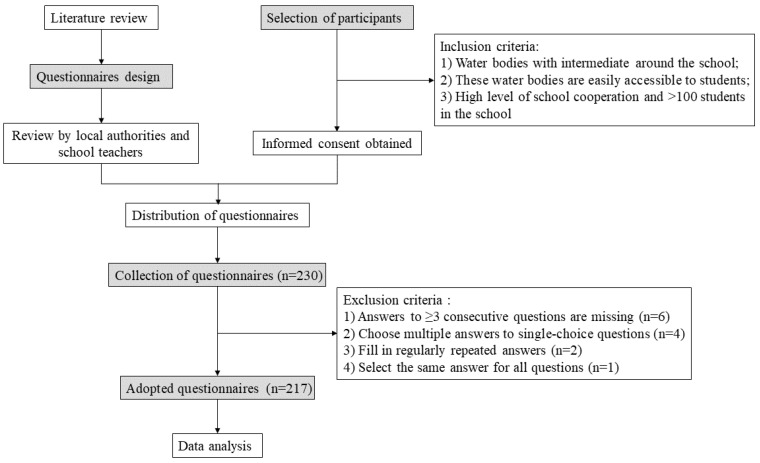
Flowchart of the study.

A face-to-face interview was conducted with a structured questionnaire in Pemba from May to September in 2021 by trained investigators at their schools, collecting baseline information and awareness of schistosomiasis from participants. Two public schools were selected in the shehia of Uwandani and Mtangni. There are permanent and temporary water bodies in the two shehias, and clustering areas of snail sites were both detected. The schools were chosen based on the following criteria: (1) there were many ponds and streams with the *Bullinus* snails in the area around school; (2) all of the students had the chance of contact with fresh water when they walked to school and back home; and (3) high level of school cooperation and sufficient number of students in the school (>100 students). Systematic sampling was used with 3 as the spacing to select subject by student ID. By the research deadline, a total of 230 students from grades 5–9 participated in the study. In total, 13 questionnaires were excluded according to the exclusion criteria, and 217 questionnaires were finally included in this study (Figure 1).

### 2.2. Questionnaire Survey

A structured questionnaire was developed in English and translated into Swahili. The content and accuracy of the questionnaire translation were independently checked by the staff from the NTD office on Pemba and the schoolteachers before conducting the survey.

The school-based questionnaire was divided into four main categories as follows:

(1) Demographic data of participants, such as age, gender, family size and parents’ education level.

(2) Knowledge (K) about schistosomiasis after the literature review [11,17,18,19], including types, signs and symptoms, transmission, prevention and control.

(3) Attitude (A) towards schistosomiasis assessed using the health brief model as a framework [20], which considered intention and perception of severity and susceptibility.

(4) Practice (P) related to schistosomiasis after a literature search [11,17,18,19], including water contact, treatment seeking, and behavioral compliance.

Additionally, questions related to COVID-19 were also included in each part of the Knowledge, Attitude and Practices (KAP) survey. The schistosomiasis knowledge section was a combination of true and false and multiple-choice questions to obtain more information from the participants. In contrast, questions about participants’ attitudes and practices toward schistosomiasis were on 5- or 3-point scales to assess the strength of attitudes toward schistosomiasis and the frequency of schistosomiasis-related practices.

### 2.3. Statistical Analysis

Epi Data 3.1 (The Epi Data Association Odense, Denmark) with dual entry and SPSS 25.0 (IBM, Armonk, NY, USA) software packages were used for statistical analyses. For the descriptive analysis, frequencies and percentages were utilized to express the categories of demographic characteristics, KAP levels and other variables. The *t*-test and chi-square test were used to examine the difference between the dependent variables (i.e., KAP score) and explanatory variables (i.e., demographic characteristics). Additionally, significant variables from univariate analysis were used as predictors in the multiple linear regression model after covariance diagnosis and exclusion of variables that affect the prediction of the results. Results from multi-factor analysis were used to calculate standardized coefficients and 95% confidence intervals (CIs) to identify the influencing factors of KAP in schistosomiasis. *p* < 0.05 was considered to indicate statistically significant differences for all tests. For the knowledge survey portion, one point was given for choosing the correct response and zero points otherwise. For the attitude and behavior portions, the scores were assigned from high to low on 5- and 3-point scales, respectively.

## 3. Results

### 3.1. General Characteristics of the Participants

A total of 217 participants responded to the questionnaires, with an average age of 15.03 years (SD: 2.14). The population was 58.99% female, and more than half (55.76%) of the children were ≤15 years of age. Moreover, 45.1% of the children belonged to a family consisting of >10 members. The parents were the main caregivers of these students [father (46.57%), mother (43.14%)]. In addition, 18.43% of the participants have been infected in the past or were infected with schistosomiasis at the time of the questionnaire, whereas 29.49% of them were unaware of their physical condition. Furthermore, 19.35% of the students reported observing blood in their urine. About 50% of the parents’ education level was below secondary level, and most of the students’ parents were farmers [father (77.88%), mother (69.12%); Table 1].

### 3.2. Knowledge about Schistosomiasis

The majority (96.31%) of the participants knew that schistosomiasis is a parasitic disease. Specifically, 91.24% of them knew about urinary schistosomiasis and 1.38% of the children could name intestinal schistosomiasis, but only 5.07% of the participants could identify both types.

A total of 217 (96.78%) children mentioned at least one sign or symptom of schistosomiasis correctly. In addition, 93.08% of the participants mentioned bloody stool or hematuria, which are typical symptoms of the intestinal and genitourinary systems. Moreover, 70.51% of the participants attributed schistosomiasis to contact with contaminated water, and only 30.41% of children were aware of snails that transmit schistosomiasis. The majority of children considered schistosomiasis to be curable (93.55%) and preventable (95.85%). With regards to prevention and the control knowledge portion of the survey, 198 children (91.24%) were able to mention at least one preventive measure (Table 2).

The mean score of knowledge was just 6.81 ± 0.98 points, and 68.70% of the children achieved an average performance (7–9 points), whereas 30.90% of the children had a poor result (<7 points).

### 3.3. Attitude towards Schistosomiasis

**Figure 2:**
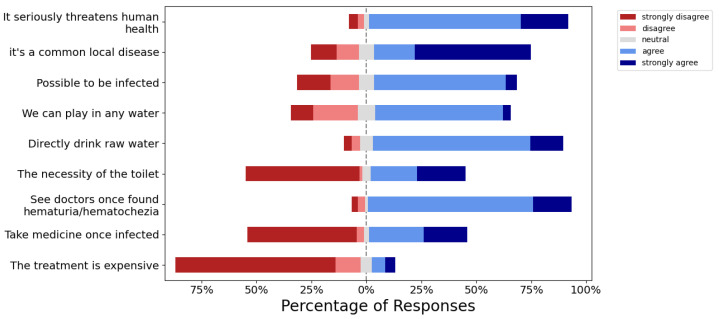
Participants’ attitudes towards schistosomiasis.

A total of 90.78% of the participants thought that schistosomiasis could pose a serious threat to human health. Approximately half of them (53.00%) strongly agreed that schistosomiasis is a common disease in their locality, and 64.98% of them considered that they might have been infected in the past or were currently infected (Figure 2). Most of the participants thought they could drink raw water directly (86.64%) and play in any type of water (61.75%). Moreover, 51.61% strongly disagreed with the necessity of having a toilet.

As for the attitude about medical treatment, although 92.63% of the participants admitted that they would go to a hospital immediately upon hematuria presentation and 84.33% of them thought the schistosomiasis treatment was not very expensive, 50.23% of the students strongly disagreed with the importance of taking medications once infected with schistosomiasis.

The participants’ attitude towards schistosomiasis scores showed the average scores of 31.64 ± 3.16 out of 47 possible points, indicating an above-moderate level of the participants’ attitude compared to 0.46% of participants who achieved a poor score.

### 3.4. Practice Related to Schistosomiasis

**Figure 3:**
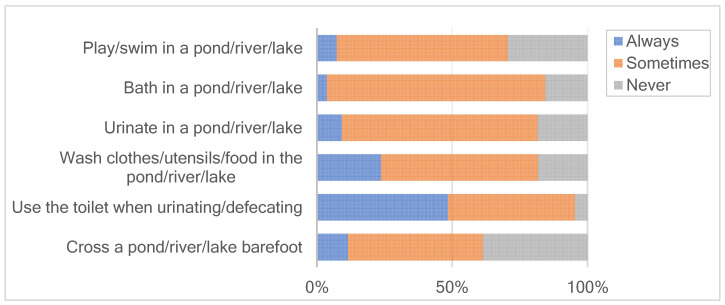
Participants’ practices related to schistosomiasis.

The risky behaviors associated with schistosomiasis infection among participants were bathing (84.33%), urinating (81.57%) and playing/swimming (70.51%) in a pond/river/lake with the confirmed presence of the *Bullinus* snail. Half of the participants and their family washed clothes/utensils/food in the pond/river/lake. Moreover, 61.3% of the participants had experiences of crossing a pond/river/lake barefoot, including individuals who chose “sometimes” or “always” as a response in the questionnaire. Of these, 11.52% indicated that they always did so barefoot. Furthermore, 84.33% of the students washed hands with tap water, whereas 5.99% did so in a river/lake/pond. Only 5.07% thought it is necessary to wash hands that were not dirty, and 1.38% of participants mentioned not using cleaning products (Figure 3). Finally, 98.16% of the children scored ≥50%, with a mean score of 13.08 ± 1.71 points.

### 3.5. Factors Associated with KAP of Schistosomiasis

Age was the only demographic factor found to be significantly associated with knowledge scores (*p* = 0.02). Different age groups showed significant differences in knowledge (*p* = 0.02) and attitude (*p* < 0.01). Participants >15 years of age scored significantly higher than the group of <15-year-olds (*p* < 0.01). Small families (<5) showed better schistosomiasis attitude and practice scores than big ones (>10). Children in families with grandparent caregivers exhibited lower scores than those with parent caregivers, demonstrating family size (*p* < 0.05) and caregivers (*p* < 0.01) to be two significant factors influencing attitude, practice and total scores. Girls performed better than boys in the practice portion of the survey (*p* < 0.01) and received higher KAP scores for schistosomiasis (*p* < 0.01). Infection status and the presence of hematuria were also two related factors in the practice portion. Mother’s education was an important factor affecting practice (*p* = 0.04), whereas mother’s occupation was a factor affecting attitude and total scores (*p* < 0.05; Table 3).

The results of multiple linear regression analyses for the factors significantly associated with schistosomiasis KAP among the participants showed that age, family size and presence of hematuria were the key factors significantly associated with the total KAP score (Table 4). Age and family size were identified as significant factors for attitude. The presence of hematuria, gender and caregiver were major influencing factors for practice. In the above univariate analysis, age was the only influencing factor, therefore, multivariate analysis was not conducted for this part.

## 4. Discussion

A total of 230 questionnaires were collected with an effective rate of 94.35% in our study. We discovered that students lacked a fundamental understanding of schistosomiasis, and despite their positive attitudes, their daily habits still required intervention. Among the key demographic factors included in this study, characters such as age and family size were considered to be the main contributing factors for the knowledge, attitude and practice of schistosomiasis in these students.

This study found that most of the children knew about urinary schistosomiasis, but they were unable to connect the type of the disease with the corresponding symptoms, and only less than a quarter could identify hematuria as a sign and symptom of urinary schistosomiasis. More than half of the children mentioned bloody stool incorrectly and chose the wrong answer to the question about “the snails that are able to transmit schistosomiasis”, indicating that knowledge of schistosomiasis was inadequate among students in Zanzibar. Lack of knowledge about schistosomiasis is a risk factor and it can be a barrier to implementing a successful control program [21]. Hence, the basic knowledge related to schistosomiasis needs to continue to be perfected and popularized to influence their practice and promote behavior change. Previous studies have emphasized the importance of school health education where students believe that school is the main source of knowledge about schistosomiasis [21]. Systematic scientific training about schistosomiasis should be provided for both school and religious educators to benefit a wide range of groups. Nowadays, it should be noted that in some African countries (including Tanzania), the COVID-19 pandemic has resulted in a learning loss of about 0.5–1 year [22] and school education is carried out predominantly face-to-face in Zanzibar. The COVID-19 crisis reminds us that establishing multiple forms of multi-channel health education is an important way to ensure disease prevention and control in the face of emergencies.

The students’ attitude towards schistosomiasis was at an above-moderate level, but what is worrying is their bad practices. Although most of the children were able to perceive the severity of and their susceptibility to schistosomiasis and could correctly describe the route of schistosomiasis infection, their high-risk behaviors, such as bathing or urinating in fresh water, still exists. Daily exposure to water was their main reason of infection. This finding signified that behavioral change is often difficult to achieve through the influence of awareness alone, and that longer time intervals are required to ensure adoption and adherence to healthier practices. The higher frequency of unhygienic behaviors may contribute to the transmission of schistosomiasis in endemic populations, similar to previous findings in Yemen [17], Kenya [18] and Mozambique [23]. Therefore, this result underscores the need for schistosomiasis control to focus on the stage of behavior change in the model of knowledge, belief and behavior.

It was observed that the risky behaviors for participants associated with schistosomiasis infection were bathing, urinating and playing/swimming in a pond/river/lake with the confirmed presence of the *Bullinus* snail, and all these behaviors are related to unsanitary water. According to research, re-infection often happens one year after treatment in high transmission settings if there has not been a change in sanitary standards and exposure patterns [21]. This indicates an urgent improvement for the access to safe and clean water to interrupt the transmission of schistosomiasis. This may also explain why more than half of the participants hold a negative attitude regarding taking medications once infected, as repeated infections caused them to question the efficacy of the medication [17,24] or the absence of drug availability [25], which could be barriers for them to make behavioral changes. Therefore, professional training in the diagnosis and treatment of schistosomiasis needs to be provided for the primary care doctors and community workers, as well as by strengthening the treatment-related policies such as MDA, to promote residents’ changes in medication behavior.

Interestingly, in addition to routine indicators such as age and gender, family size was noticed as another significant predictor of attitude and practice in children’s perception of schistosomiasis. Participants with a smaller family had a more positive attitude and performed better. Members from the same family use the same water source and have similar behaviors in their daily life. Limited water sanitation will keep all household members at risk of infection and thus suffer from high incidence of diseases. However, interventions that target only certain members of the family, such as students, are insufficient. Family-based interventions may ensure that each individual could be included. Therefore, a community-directed intervention strategy may be an effective strategy for addressing schistosomiasis health interventions, as familiarity of community health workers with households, their knowledge of intervention area and effective community mobilization are important complements to school health education and represent other opportunities for MDA implementation [26]. Therefore, for the prevention and control of schistosomiasis, community-oriented intervention [26,27] combined with school-based approaches [6] will be more successful with the cooperation of different government departments. Some limitations should be noted in this study. Although we obtained a very representative group of participants in this study, the sample should be expanded to the other countries in Africa and more details relating to the KAP of students could be studied in subsequent projects. Moreover, due to the infection status of participants being based on their own descriptions, they may exaggerate or reported socially desirable answers, and the results could not reveal the details about past infection and current infection. Therefore, in follow-up studies, we will conduct urine detection of schistosomiasis while collecting the questionnaire, and in subsequent projects, we will also send questionnaires to students’ parents or guardians, which is a reliable way to gauge the family awareness and complete the missing information from the students, such as the family status and their physical condition. In addition, the questionnaire was designed with objective multiple-choice questions, which led to the limited content of respondents’ answers. Subjective questions should be appropriately added in the follow-up work, and more detailed and in-depth information could be explored.

## 5. Conclusions

Despite the high awareness of schistosomiasis among students, there was a gap among the students regarding the symptoms of schistosomiasis, attitude toward taking medication after infection, and unsanitary water contact behaviors. Therefore, health education should be strengthened to improve students’ awareness of schistosomiasis and prevent unsanitary water contact behavior in families, schools, churches and other public spaces, with special emphasis on the routes of transmission and the consequences of untreated diseases. This should run side by side with other control measures carried out by health authorities. Comprehensive prevention and control measures such as improving water sanitation and enhancing preventive chemotherapy are part of successful schistosomiasis campaigns. In addition, family size is an interesting and important influencing factors in our study, which provides a new perspective for schistosomiasis prevention and control. Family-based intervention in the communities may be a new way to achieve the goal of schistosomiasis in the NTD Roadmap 2030.

## Figures and Tables

**Table 1:** Participant characteristics.

	Characteristics	N (%)
Age (years) (217)	≤15	121 (55.76)
	>15	96 (44.24)
Sex (217)	Male	89 (41.01)
	Female	128 (58.99)
Residence (217)	Mtangani	130 (59.91)
	Uwandani	87 (40.09)
Grade (217)	Primary	73 (33.64)
	Secondary	144 (66.36)
Family size (173)	<5	39 (22.54)
	5~10	56 (32.37)
	>10	78 (45.09)
Caregiver (204)	Father	95 (46.57)
	Mother	88 (43.14)
	Grandparents	18 (8.82)
	Others	3 (1.47)
Infection status (217)	Yes	40 (18.43)
	No	113 (52.07)
	No idea	64 (29.49)
Father’s education (217)	Illiteracy	36 (16.59)
	Primary	68 (31.34)
	Secondary and higher	65 (29.95)
	No idea	48 (22.12)
Mother’s education (217)	Illiteracy	40 (18.43)
	Primary	58 (26.73)
	Secondary and higher	83 (38.25)
	No idea	36 (16.59)
Father’s occupation (217)	Farmer	169 (77.88)
	Government staff	16 (7.37)
	Others	32 (14.95)
Mother’s occupation (217)	Farmer	150 (69.12)
	Government staff	12 (5.53)
	Others	55 (25.35)
Blood in urine or not (217)	Yes	42 (19.35)
	No	175 (80.65)

Note: N, number of participants.

**Table 2:** Participants’ knowledge about schistosomiasis.

Variable	N (%)
Schistosomiasis is one kind of parasites diseases (217)	
True	209(96.31)
False	8(3.69)
What types of schistosomiases are included (217)	
Intestinal schistosomiasis	3(1.38)
Urinary schistosomiasis	198(91.24)
Both	11(5.07)
No idea	5(2.30)
What are the signs and symptoms of schistosomiasis (217)	
Abdominal pain	2(0.92)
Diarrhea	4(1.84)
Bloody stool	149(68.66)
Hematuria	53(24.42)
Vomiting	3(1.38)
Weakness	1(0.46)
Odynuria	1(0.46)
No idea	4(1.84)
How can a person develop schistosomiasis (217)	
Contact with contaminated water	153(70.51)
Eating raw or undercooked fish/meat	4(1.84)
Eating unwashed vegetables	8(3.69)
Close contact with schistosomiasis	11(5.07)
No idea	42(19.35)
Snails are able to transmit schistosomiasis (217)	
True	66(30.41)
False	151(69.59)
Schistosomiasis is curable (217)	
True	203(93.55)
False	14(6.45)
Schistosomiasis is preventable (217)	
True	208(95.85)
False	9(4.15)
How do the controls and prevention work in your locality (217)	
Snail extermination	26(11.98)
Setting up voice prompters	156(71.89)
Building water pipes	16(7.37)
Science brochure	8(3.69)
No idea	19(8.76)

Note: N, number of participants.

**Table 3:** Demographic determinants of participants’ KAP scores related to schistosomiasis.

Variable	Knowledge		Attitude		Practice		Total Score	
	Mean (SD)	*p*	Mean (SD)	*p*	Mean (SD)	*p*	Mean (SD)	*p*
Age		0.02		<0.01		0.07		<0.01
≤15	6.67(1.05)		30.88(2.64)		12.89(1.76)		50.44(3.28)	
>15	6.99(0.85)		32.59(3.50)		13.31(1.61)		52.90(4.10)	
Sex		0.58		0.81		<0.01		<0.01
Male	6.76(1.17)		31.57(3.12)		12.25(1.67)		50.58(3.67)	
Female	6.84(0.83)		31.68(3.20)		13.66(1.48)		52.18(3.86)	
Residence		0.08		<0.01		0.38		<0.01
Mtangni	6.72(1.02)		30.93(2.72)		15.78(1.89)		53.43(3.54)	
Uwandani	6.95(0.90)		32.69(3.48)		16.02(2.01)		55.67(4.20)	
Family size		0.51		0.04		0.03		0.01
<5	6.85(0.88)		32.46(2.99)		13.72(1.43)		53.03(3.67)	
5–10	6.98(0.77)		31.88(3.36)		12.77(1.96)		51.63(3.98)	
>10	6.78(1.17)		30.92(3.18)		13.04(1.73)		50.74(3.81)	
Caregiver		0.73		<0.01		<0.01		<0.01
Father	6.86(0.79)		31.05(2.46)		13.26(1.78)		51.18(3.24)	
Mother	6.80(1.03)		32.59(3.23)		13.36(1.50)		52.75(3.69)	
Grandparents	6.56(1.58)		30.11(4.42)		11.78(1.52)		48.44(5.10)	
Others	6.88(0.89)		31.56(3.58)		11.88(1.50)		54.00(1.00)	
Infection status		0.59		0.89		<0.01		0.03
Yes	6.93(0.66)		31.60(2.88)		11.58(1.60)		50.10(3.49)	
No	6.80(0.91)		31.56(3.06)		13.60(1.50)		51.96(3.66)	
Unclear	6.77(1.24)		31.80(3.52)		13.09(1.57)		51.66(4.23)	
Father’s education		0.69		0.13		0.45		0.56
Illiteracy	6.83(0.56)		31.58(2.14)		13.08(1.96)		51.50(3.15)	
Primary	6.81(1.20)		31.88(3.66)		12.99(1.59)		51.68(4.40)	
Secondary and higher	6.82(0.88)		31.94(3.68)		12.92(1.58)		51.68(4.24)	
No idea	6.79(1.03)		30.92(2.04)		13.42(1.82)		51.13(2.94)	
Mother’s education		0.72		0.26		0.04		0.76
Illiteracy	6.83(0.96)		32.03(3.65)		13.03(1.63)		51.88(3.58)	
Primary	6.71(0.77)		31.67(3.37)		13.41(1.59)		51.79(4.25)	
Secondary and higher	6.82(1.10)		31.70(3.18)		12.70(1.74)		51.22(4.10)	
No idea	6.94(1.04)		31.00(2.00)		13.47(1.77)		51.42(2.84)	
Father’s occupation		0.25		0.13		0.68		0.12
Farmer	6.87(1.00)		31.62(2.86)		13.05(1.71)		51.54(3.50)	
Government staff	6.63(1.09)		33.00(5.90)		13.44(1.41)		53.06(7.27)	
Others	6.59(0.76)		31.03(2.65)		13.06(1.83)		50.69(3.13)	
Mother’s occupation		0.12		<0.01		0.43		0.03
Farmer	6.90(1.02)		32.01(3.07)		12.98(1.67)		51.89(3.63)	
Government staff	6.75(0.97)		32.00(6.15)		13.17(1.47)		51.92(8.06)	
Others	6.58(0.83)		30.53(2.12)		13.33(1.85)		50.44(2.87)	
Blood in urine or not		0.87		0.97		<0.01		<0.01
Yes	6.83(0.73)		31.62(2.91)		11.33(1.68)		49.79(3.89)	
No	6.81(1.03)		31.64(3.22)		13.50(1.43)		51.94(3.74)	

**Table 4:** Multivariate analysis of factors associated with schistosomiasis KAP.

Variable		Unstandardized Coefficients		Standardized Coefficients		*p*	95%CI
		*β*	*Se*	*β*	*t*		
Attitude	Constant	31.32	1.08		29.05	<0.01	(29.19, 33.45)
	Age	1.24	0.50	0.19	2.48	0.01	(0.25, 2.22)
	Family size	−0.64	0.31	−0.16	−2.07	0.04	(−1.26, −0.03)
Practice	Constant	8.05	0.62		12.90	<0.01	(6.81, 9.28)
	Hematuria	2.16	0.28	0.48	7.78	<0.01	(1.61, 2.71)
	Sex	1.15	0.22	0.32	5.22	<0.01	(0.71, 1.58)
	Caregiver	−0.34	0.15	−0.14	−2.29	0.02	(−0.64, −0.05)
Total score	Constant	47.67	1.77		26.98	<0.01	(44.18, 51.16)
	Age	2.09	0.58	0.27	3.64	<0.01	(0.96, 3.23)
	Family size	−0.90	0.36	−0.19	−2.51	0.01	(−1.60, −0.19)
	Hematuria	1.67	0.73	0.17	2.29	0.02	(0.23, 3.12)

## Data Availability

The data presented in this study are available on request from the corresponding author. The data are not publicly available because of privacy issues.
